# Visualisation in imaging mass spectrometry using the minimum noise fraction transform

**DOI:** 10.1186/1756-0500-5-419

**Published:** 2012-08-07

**Authors:** Glenn Stone, David Clifford, Johan OR Gustafsson, Shaun R McColl, Peter Hoffmann

**Affiliations:** 1 School of Computing, Engineering and Mathematics, University of Western Sydney, Sydney, New South Wales, Australia; 2 Division of Mathematics, Informatics and Statistics, CSIRO, Brisbane, Queensland, Australia; 3 Adelaide Proteomics Centre, School of Molecular and Biomedical Science, The University of Adelaide, Adelaide, South Australia, Australia

**Keywords:** Dimension reduction, MALDI imaging mass spectrometry, Image processing

## Abstract

**Background:**

Imaging Mass Spectrometry (IMS) provides a means to measure the spatial distribution of biochemical features on the surface of a sectioned tissue sample. IMS datasets are typically huge and visualisation and subsequent analysis can be challenging. Principal component analysis (PCA) is one popular data reduction technique that has been used and we propose another; the minimum noise fraction (MNF) transform which is popular in remote sensing.

**Findings:**

The MNF transform is able to extract spatially coherent information from IMS data. The MNF transform is implemented through an R-package which is available together with example data from http://staﬀ.scm.uws.edu.au/∼glenn/∖#Software.

**Conclusions:**

In our example, the MNF transform was able to find additional images of interest. The extracted information forms a useful basis for subsequent analyses.

## Background

Imaging Mass Spectrometry (IMS) provides a means to measure the spatial distribution of drug metabolite, lipid, peptide and protein features on the surface of a sectioned tissue sample (see [1] and references therein). Typically, IMS methods utilise freshly frozen sections of tissue mounted onto conductive slides. These are coated with matrix followed by MALDI-ToF/ToF spectra acquisition at anywhere from hundreds to thousands of positions across a tissue, the spatial locations of which are annotated. For example, a section of coronal murine midbrain can generate more than ∼2000 spectra. Data acquisition at 0.1 GS/s over an m/z range 1000-26000 yields individual mass spectra with more than 11,000 plotted points. The resulting data set is enormous and thus difficult to process, visualise and analyse effectively.

**Figure 1:**
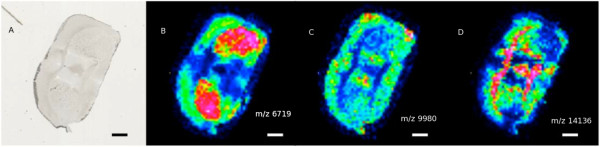
**Image(A)of coronal murine midbrain section, and ion intensity maps at m/z of 6719(B), 9980(C) and 14136(D).** m/z are approximate, from flexImaging V2.1. Scale bar is 1 mm.

The data can be thought of in two ways, firstly a set of mass spectra acquired at a spatial array of spots, and secondly as a *stack* of ion intensity maps, each map being akin to a low resolution image. Software such as Biomap and flexImaging (Bruker Daltonics) view IMS data as ion intensity maps and include features such as data normalisation and noise spectra exclusion (see Figure 1). However the choice of ion intensity maps to view is largely user driven and images are noisy. Further data analysis using external software packages is possible, for example, (ClinProTools for principal component analysis (PCA), hierarchical clustering (HC) of spectra, or spectral model generation [2-5]. Other analysis techniques used on IMS data include kriging of ion intensity maps [6] and supervised classification methods, for example, random forests [7].

Current methods typically use spectral features, not spatial information, to guide analysis. Hence the predominance of PCA and HC type approaches. We propose the use of the minimum noise fraction (MNF) transform [8] to, firstly, determine the most interesting spatial representations of IMS data, and secondly, form the basis of data reduction for subsequent analysis. The MNF transform has previously been used on hyper-spectral images of tissue samples [9] but this is the first use of such a technique on IMS data.

## Findings

### Principal Components Analysis

Principal Components Analysis (PCA) treats the IMS data as a collection of spectra. Therefore, in PCA, the spatial structure of the spots is not relevant and so the data can be represented as a matrix $풵=\{Z_{\mathrm{ik}}\}$ where *i*=1,…,*n* ranges over the spots on the tissue and *k*=1,…,*p* ranges over the mass charge ratios in the mass spectrum. Let *Z*={_*Zk*_} be a typical mass spectrum. PCA seeks linear combinations of intensities over the mass charge ratios that maximizes variance. That is, the first principal component is defined by a vector *a*={_*ak*_} with *a* chosen so that Var(^*at*^*Z*) is maximised. Second and subsequent principal components maximize variance subject to being uncorrelated with all previous principal components.

If _*ΣZ*_ is the covariance matrix of the mass spectra, that is, the (_*k*1_,_*k*2_) entry is the covariance of the ion intensity measured at the *k*_1_-th m/z ratio and the ion intensity measured at the *k*_2_-th m/z ratio, then the first principal component maximises $a^{t}\Sigma_{Z}a$ subject to a suitable scale constraint such as ^*at*^*a*=1. Generally, _*ΣZ*_is unknown so is estimated using the sample covariance matrix *S*_*Z*_ given by

(1) $${\left(S_{Z}\right)}_{k_{1}k_{2}}=\frac{1}{n-1}\underset{i}{\sum }\left(Z_{ik_{1}}-{\bar{Z}}_{\cdot k_{1}}\right)\left(Z_{ik_{2}}-{\bar{Z}}_{\cdot k_{2}}\right)$$

where

(2) $${\bar{Z}}_{\cdot k}=\frac{1}{n}\underset{i}{\sum }Z_{\mathrm{ik}}$$

It should be noted that the mass spectra are unlikely to form a set of independent observations since spatially close spectra will likely be correlated.

### The MNF transform

PCA makes no use of the spatial structure of the observed mass spectra. The Minimum Noise Fraction transform uses a simple model to allow the spatial structure to influence the analysis. Here we modify the notation to emphasise the spatial aspect; let *Z*(*x*) be the mass spectrum at spatial location *x*. In our case, *x* will be a spot on the tissue section indexed by a horizontal and a vertical coordinate. A possible model for *Z*(*x*) is

(3) Z(x)=M(x)+N(x)

where *M*(*x*) represents the *signal* at *x* and *N*(*x*) is the *noise* at *x*.

This is to be interpreted as “the mass spectrum at spot *x* is composed of a spatial signal mass spectrum plus a noise mass spectrum”. We assume the signal and noise components to be independent, and the noise component to have low spatial covariance. The signal component would likely have high spatial covariance. Both components would still have a covariance between intensities at differing mass charge ratios, represented by covariance matrices _*ΣM*_ and _*ΣN*_.

The MNF transform seeks linear combinations of intensities over the mass charge ratios that maximizes *signal to noise ratio* (SNR). That is, the first MNF band is defined by a vector *a*={_*ak*_} with *a* chosen so that SNR=Var(^*at*^*M*)/Var(^*at*^*N*) is maximised. Replacing the variances by expressions in terms of the covariance matrices we see that;

(4) $$\text{SNR}=\frac{a^{t}\Sigma_{M}a}{a^{t}\Sigma_{N}a}$$

In PCA we need an estimate for _*ΣZ*_, whereas for the MNF we need estimates of _*ΣM*_and _*ΣN*_. These are not as straight-forward to obtain as in PCA, since the signal *M* and noise *N* components are not directly observed. However, by noting that (by independence) _*ΣZ*_=_*ΣM*_ + _*ΣN*_we see that the SNR is maximised when the following ratio is maximised,

(5) $$\frac{a^{t}\Sigma_{Z}a}{a^{t}\Sigma_{N}a}$$

Thus only an estimate for _*ΣN*_ is required. In reality, only an estimate of _*ΣN*_*or*_*ΣM*_ is required, and we find it easiest to estimate the former.

Green et al. [8] propose a *shift difference* method to estimate _*ΣN*_ and Berman et al. [9] propose using the covariance of residuals from a local quadratic fit. In the latter case, a quadratic function is fit to a 3×3 neighbourhood of each spot for each mass charge ratio, and a residual computed at the spot. This produces a set of pseudo-residual data and the sample covariance of this used as the estimate. These original applications of the MNF transform are based on hyperspectral images where the spots are very close together. Here the spots from which MS spectra are collected are somewhat separated. For this reason we have used a simpler local linear fit, based on the ideas of [10], which is similar to using a symmetrical set of shift differences. Using the simpler approach places less reliance on spots that are further apart. Although there is scope to investigate other approaches, preliminary work shows little difference when a quadratic signal fit is used in this case.

Each spot (except edge spots) has two horizontal and two vertical neighbours. Averaging these four values gives the prediction of a local linear fit at the central spot, from which a pseudo-residual can be derived. Since the spots are on a regular grid, this corresponds to the residual from a local linear fit to the four neighbouring spots. This procedure produces a set of pseudo-residuals (one for each spot at each mass charge ratio, subject to simple modification at edge spots) from which the sample noise covariance matrix *S*_*N*_ can be formed. We use this as the estimate of _*ΣN*_.

### Implementation

PCA corresponds to the maximisation of $a^{t}S_{Z}a$ subject to a scale constraint such as ^*at*^*a*=1. Lagrange multipliers can be used to show that at the maximum, *a* is the eigenvector of *S*_*Z*_ corresponding to the largest eigenvalue. Subsequent principal components are defined by eigenvectors corresponding to subsequent eigenvalues. A similar argument shows that the *a* which maximises the ratio

(6) $$\frac{a^{t}S_{Z}a}{a^{t}S_{N}a}$$

satisfies

(7) $$S_{Z}a=\lambda S_{N}a$$

This is a *generalised eigenproblem* (see [11] for example).

For both PCA and MNF the uses of all mass charge ratios would produce sample covariance matrices that are extremely large, so firstly some pre-filtering is used. In PCA, this is often a peak identification method, or selection by taking all the mass charge ratios for which the intensity exceeds some threshold (in some or all spots). For the MNF transform, we use only those mass charge ratios whose SNR values exceed a threshold. This SNR corresponds to the ratio of diagonal entries in *S*_*Z*_ and *S*_*N*_. The threshold is chosen so that the matrices are of a manageable size.

Our implementation uses the LAPACK [12] routines for generalised eigenproblem interfaced to the R system for statistical programming [13]. All aspects of this process are automated in our code. The only manual interventions required have to do with pre-filtering of the signals and the choice of the number of bands for subsequent analysis. Both of these manual interventions are required by PCA also.

### Results

We demonstrate the method using a section of 10 *μ*m coronal murine midbrain. The section was desiccated for 30 minutes followed by washing in 70% and 100% EtOH for 30 seconds each, and subsequently desiccated until use. 20 mg/mL of 2,5-dihydroxybenzoic acid in 50% MeOH and 0.2% TFA matrix was deposited using 3 phases on an ImagePrep station. Mass spectrometry analysis was carried out on an UltraFlex III MALDI-TOF/TOF machine in linear positive ion mode. ClinProT calibrants (1:20 dilution with matrix) were used to calibrate the instrument. Data acquisition used flexControl V3.3, with 300 shots taken at each spot and summed. Mass spectra were acquired in m/z range 1000–26000 at a rate of 0.1 GS/s. Figure 1 shows the image of the section (A) and flexImaging (Bruker Daltonics) generated ion intensity maps for three m/z ratios (B–D).

The processed data consists of intensities at 11280 mass charge ratios, repeated across a grid of 2012 spots over the tissue slice. The data were first logged and then background corrected by using a 5-knot robust spline fit to estimate baseline. Pre-filtering of mass charge ratios was carried by thresholding intensities (in the case of PCA) or SNRs (in the case of MNF) so that 650 were retained. PCA and MNF transforms were computed. This means that PCA operated on the 650 mass charge ratios with the highest intensity, whereas MNF used the 650 mass charge ratios with the highest estimated signal to noise. The choice of 650 data points stems from trial and error and a pragmatic desire to use manageable covariance matrices.

**Figure 2:**
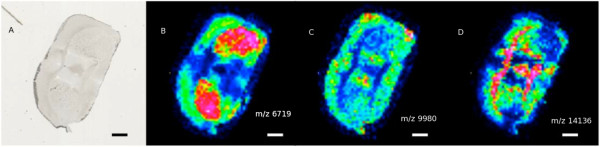
The first six principal component images of a coronal murine midbrain section.

Figure 2 shows the first six principal component images of the data, there appears to be three images with significant spatial structure, and the remaining three appear to be noise.

**Figure 3:**
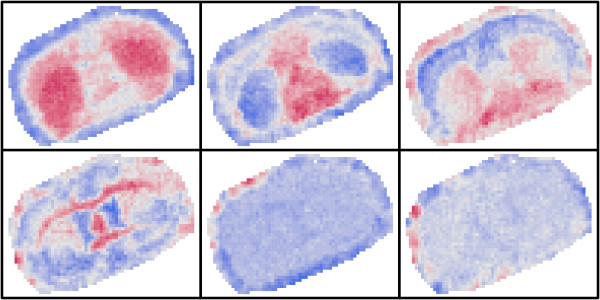
The first six MNF transformed images of a coronal murine midbrain section.

Figure 3 shows the first six MNF bands. There are four images with clear spatial structure, so the MNF transform has been able to extract further information.

### Subsequent Analysis

Deininger et al. [3] show the use of the principal components in hierarchical clustering, and this can also be done with the MNF bands. Hierarchical clustering is useful for identifying regions of the tissue with relatively homogenous properties. Using the PCA or MNF bands significantly reduces the computational complexity of clustering without overly reducing its usefulness.

**Figure 4:**
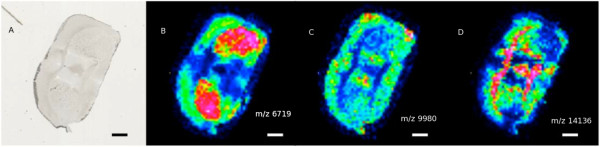
**The results of clustering using the first four MNF bands.(A)** the clustering of spots (spots in the same cluster have the same colour), **(B)** the average (background corrected) mass spectrum for each cluster

Taking the first four MNF band images, and applying hierarchical clustering we can determine seven clusters. Figure 4 shows the results. Panel A shows the spots coloured according to cluster and panel B shows the average mass spectrum for the spots in each cluster.

As with PCA, the choice of the number of MNF bands to use in subsequent analysis (such as hierarchical clustering) is somewhat ad-hoc and depends on the form of such analysis. For clustering and classification there are many methods for choosing the number of features but we regard this as a topic for further research.

In this instance the number of components chosen (six) was primarily chosen for convenience and subjective reasons. The 5th and 6th PCA plots still show some faint internal structure, whereas subsequent ones do not (not shown). So we use 6 components for both PCA and MNF for consistency.

More generally the number of PCA components *can* be chosen using percent total variation explained arguments. In this approach, the sum of the eigenvalues for the chosen components divided by the sum of *all* the eigenvalues, converted to a percentage, is considered. A threshold percent (eg. 80 or 90%) is then chosen and the number of principal components fixed at that which first exceeds the threshold. It is not so easy to apply this technique for MNF as the eigenvalues represent signal to noise ratios and as such are not additive. However, since they are signal to noise ratios, they are scale-free and can be subject to thresholds themselves ie. take all components with eigenvalue (signal-to-noise ratio) greater than a threshold. Examples of such a threshold might be one, ie. signal and noise are approximately equal.

## Conclusion

We have shown that the minimum noise fraction transform is a potent addition to the suite of analysis tools available for the analysis of Imaging Mass Spectrometry data. Like PCA, we have further demonstrated that the MNF bands generated can be used as summaries of the mass spectra to analyse the spatial characteristics of a tissue slice. We regard the MNF transform as providing a useful alternative to PCA in Imaging Mass Spectrometry. Its defining feature is that is uses estimates of spatial signal to noise ratio to sequentially define bands whereas PCA uses only total variation (signal plus noise).

Both PCA and MNF are computationally efficient when compared to the data acquisiton and preprocessing steps involved. In our implementation, all code was written in R and C and is therefore platform independent. However, the flexImaging provided data in a proprietary format that required the use of a Windows only proprietary tool (CompassXport). We have successfully used emulation software on Linux and Mac OS X based systems to run this tool.

## Availability and requirements

**Project Name:** Computing Minimum Noise Fraction Transforms of Imaging Mass Spectrometry Data;**Project Home:**http://staﬀ.scm.uws.edu.au/∼glenn/#Software;**Operating Systems:** MNF code is in R and C and is compatible with Windows, Mac, and Linux;**Programming Language:** R, http://cran.r-project.org and C;**Other Requirements:** caMassClass,[14]; Data Acquistion and conversion software (flexImaging/CompassXport);**License** GPL-2;**Restrictions to use by non-academics:** none;

## Availability of supporting data

The software and supporting data are available for download from the project home at http://staﬀ.scm.uws.edu.au/∼glenn/#Software.

## Competing interests

The authors declare that they have no competing interests.

## Author’s contributions

GS and DC concieved the statistical approach. DC implemented the analysis. GS drafted the manuscript. JORG, SRM and PH developed the protocols, and obtained, prepared and processed the samples. All authors read and approved the final manuscript.

## Funding

GS was employed by CSIRO when much of this work was carried out. The Adelaide Proteomics Centre was partially funded by Bioplatforms Australia and an NHMRC equipment grant.
